# Class IA PI3K regulatory subunits: p110-independent roles and structures

**DOI:** 10.1042/BST20190845

**Published:** 2020-07-17

**Authors:** Millie Fox, Helen R. Mott, Darerca Owen

**Affiliations:** Department of Biochemistry, University of Cambridge, 80 Tennis Court Road, Cambridge CB2 1GA, U.K.

**Keywords:** cytoskeleton, nuclear functions, p85, phosphoinositide 3-kinase, receptor trafficking, regulatory dimers

## Abstract

The phosphatidylinositol 3-kinase (PI3K) pathway is a critical regulator of many cellular processes including cell survival, growth, proliferation and motility. Not surprisingly therefore, the PI3K pathway is one of the most frequently mutated pathways in human cancers. In addition to their canonical role as part of the PI3K holoenzyme, the class IA PI3K regulatory subunits undertake critical functions independent of PI3K. The PI3K regulatory subunits exist in excess over the p110 catalytic subunits and therefore free in the cell. p110-independent p85 is unstable and exists in a monomer-dimer equilibrium. Two conformations of dimeric p85 have been reported that are mediated by N-terminal and C-terminal protein domain interactions, respectively. The role of p110-independent p85 is under investigation and it has been found to perform critical adaptor functions, sequestering or influencing compartmentalisation of key signalling proteins. Free p85 has roles in glucose homeostasis, cellular stress pathways, receptor trafficking and cell migration. As a regulator of fundamental pathways, the amount of p110-independent p85 in the cell is critical. Factors that influence the monomer-dimer equilibrium of p110-independent p85 offer additional control over this system, disruption to which likely results in disease. Here we review the current knowledge of the structure and functions of p110-independent class IA PI3K regulatory subunits.

## Introduction

**Figure 1.**
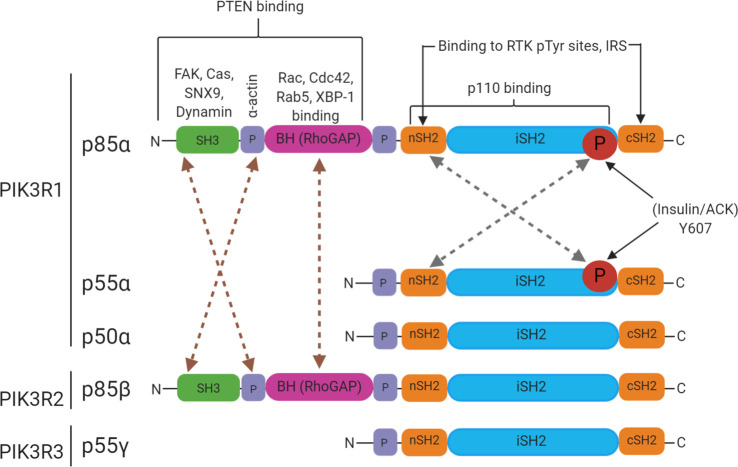
Domain architecture of the Class IA PI3K regulatory subunits. SH3: Src homology-3 domain, P: proline-rich sequences, BH(RhoGAP): domain with sequence homology to the Rho family GTPase-activating domain of Bcr, SH2: Src homology-2 domain, nSH2: N-terminal SH2 domain, cSH2: C-terminal SH2 domain, iSH2: inter-SH2 region. Dashed lines between domains refer to the interactions that would be occur in regulatory subunit dimers. Brown lines show interactions between the N-terminal domains (SH3, P and BH) that are formed in the N-terminal dimers. Although these interactions are shown between p85α and p85β, p85α/p85α and p85β/p85β interactions are also applicable. Grey dashed lines show the interactions between the nSH2 and phosphotyrosine at position 607 in the iSH2 region that drives C-terminal dimers. These interactions are shown between p85α and p55α but dimers such as p85α/p85α and p55α/p55α can also form.

Phosphatidylinositol 3-kinases (PI3Ks) are a family of lipid kinases that catalyse the transfer of the γ-phosphate group of ATP to the D3 position of phosphoinositides. PI3Ks are grouped into three classes based on their substrate specificity (Table 1). Class IA PI3Ks are obligate heterodimers composed of a p110 catalytic subunit and a p85 regulatory subunit. There are five different isoforms of the regulatory subunits: p85α, p85β, p55α, p50α and p55γ, but here the term p85 is used generically to refer to all Class IA PI3K regulatory subunits when the isoform is otherwise not stated. The p85α/p110α heterodimer has been intensely investigated and its fundamental regulatory mechanisms have been identified. The regulatory subunit stabilises the overall conformation of the catalytic subunit and inhibits its lipid kinase activity. The domain architecture of the class IA PI3K regulatory subunits is shown in Figure 1. The C-terminal half of all regulatory subunit isoforms comprises two SH2 domains separated by an inter-SH2 domain, the major site responsible for binding the p110 catalytic subunit [1]. The nSH2 domain of p85 also contributes to regulatory contacts with p110α [2], while for p110β and p110δ, catalytic activity is restrained by all three p85 domain contacts: the cSH2, nSH2 and iSH2 [3,4]. Upon receptor tyrosine kinase activation, the p85/p110 complex is recruited to phosphotyrosine residues in the activated receptor via the p85 SH2 domains. This alleviates the inhibitory contacts with the p110 catalytic subunit, while also bringing p110 into close proximity with its lipid substrates. Class IA PI3Ks phosphorylate phosphoinositide 4,5-bisphosphate (PIP_2_) leading to the production of phosphoinositide 3,4,5-triphosphate (PIP_3_), which serves as a major second messenger molecule, recruiting effectors which then activate signalling cascades [1,5–8]. The PI3K pathway is a pivotal regulator of many cellular processes and is essential in eukaryotic cells. Activities such as cell survival, proliferation, cytoskeletal remodelling, vesicular trafficking and motility are governed by the PI3K pathway [9].

**Table 1:** Classification of mammalian PI3Ks

Class	Catalytic subunit	Regulatory subunit	Lipid Substrate
IA	p110α, p110β, p110δ	p85α, p85β, p55α, p55γ, p50α	PI, PI4P, PIP_2_
IB	p110γ	p101, p84/87	PI, PI4P, PIP_2_
II	PI3K-C2α, -C2β, -C2γ		PI PI4P
III	Vps34	P150	PI

PI4P: Phosphatidylinositol 4-phosphate, PIP_2_: phosphatidylinositol (4,5)-bisphosphate, VPS34: vesicular protein sorting 34. PI: Phosphatidylinositol.

**Figure 2.**
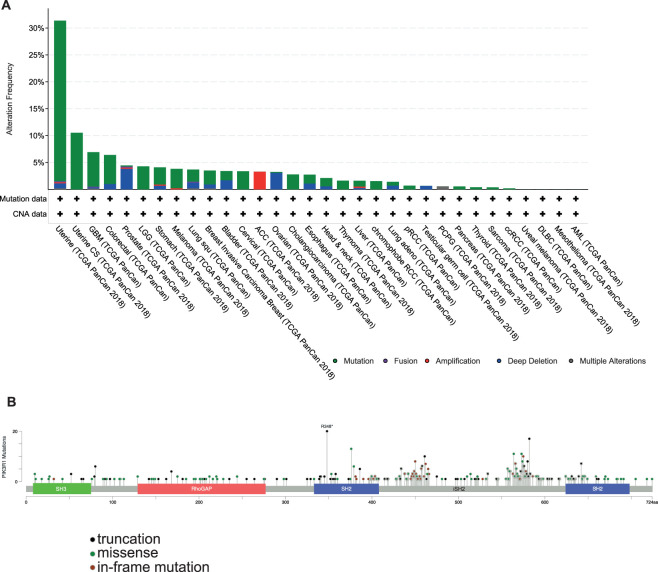
Frequency and location of PIK3R1 mutations in cancer. (**A**) The frequency of changes in the *PIK3R1* gene by mutation (green), fusion (purple), amplification (red), deep deletion (blue) or multiple alterations (grey) displayed across all cancer types. (**B**) *PIK3R1* mutations mapped onto domain architecture. Circles coloured with respect to corresponding mutation types: black, truncating mutations; green, missense mutations; brown, in-frame mutations. In case of different mutation types at a single position, the colour of the circle is determined with respect to the most frequent mutation type. Figure generated using cBioportal [16,17].

For the class IA PI3Ks the three catalytic subunits are encoded by three separate genes: *PIK3CA, PIK3CB, PIK3CD*. Mutations in *PIK3CA* are commonly found in solid human cancers and also in benign overgrowth syndromes known collectively as *PIK3CA*-related overgrowth spectrum (PROS) [10,11]. In contrast, there are three genes (*PIK3R1, PIK3R2, PIK3R3*) encoding the five different regulatory subunits. *PIK3R1* codes for p85α, p50α and p55α, *PIK3R2* for p85β and *PIK3R3* for p55γ. p85α, the most abundant isoform in normal tissues is a tumour suppressor and its expression is often reduced in cancer [12]. In contrast, p85β also ubiquitous in cells, is frequently overexpressed in cancers and is considered an oncogene [13]. In comparison with *PIK3CA*, mutations in *PIK3R1* are less common but have been found in various cancers including endometrial, colorectal, breast and pancreatic [14,15]. Mutations are the most common genetic alteration in *PIK3R1* and are most commonly found in uterine cancers, 30% of which have a *PIK3R1* mutation (Figure 2A) [16,17]. Somatic mutations are found throughout *PIK3R1* but cluster within the iSH2 domain (Figure 2B), disrupting the inhibitory contacts to p110 and resulting in PI3K pathway activation [14,15,18–20]. Contrastingly, *PIK3R1* mutants have also been reported that do not bind p110 and do not result in Akt activation [15]. These mutations affect structurally important domains, preferentially occur with p110α mutations and are found at high frequency in endometrial and colorectal cancers, suggesting important roles for p110-independent p85 [15].

In addition to its canonical function in regulating the PI3K catalytic subunit many p110-independent functions of p85 have now been reported. To date, these roles are predominantly adaptor functions, localising signalling proteins and coordinating their function. Notably many of these roles exist in cellular stress-induced pathways e.g. in the unfolded protein response [21–23], p53-mediated senescence [24,25] and the insulin response. In fact the p85 proteins play a dual role in mediating glucose homeostasis, as part of the PI3K heterodimer and independently of p110 [26]. p110-independent p85 also plays crucial roles in mediating cytoskeletal changes [27,28] as well as endocytic trafficking [29].

## A dynamic equilibrium between monomeric and dimeric p85 regulates signalling through the PI3K pathway

### Free p85 sets the threshold for class IA PI3K activation

PI3K signalling results in activation of multiple signalling pathways. It is therefore not surprising that overactivation of this pathway has been identified in a range of diseases including cancer, diabetes, obesity and autoimmunity. The molecular balance between the regulatory and catalytic subunits of PI3K is critical in maintaining activity. The p85α subunit is more abundant than p110α and therefore exists independently of p110 [12]. Absolute quantification of the PI3K subunits found p85s to be in molar-excess of p110s, both in MEFs (p85β by 20%) and liver (p85α by 30%) [30]. Free p85 performs a key inhibitory function in the PI3K pathway by competing with the p85–p110 complex for receptor binding. However complete depletion of p85α in *PIK3R1*^-/-^ homozygous MEFS caused a significant decrease in PI3K activity, attributed to a decrease in both p85 and p110. Depletion of p85 reduces p110 levels, as p85 is an absolute requirement for p110 stability. However in *PIK3R1*^-/+^ heterozygous MEFS, the 50% reduction in p85α led to enhanced PI3K responses, owing to decreased inhibition by free p85 [12]. In fact, p110-free p85 has a higher affinity and enhanced co-operative binding to tyrosine phosphorylated proteins in comparison with the p85–p110 complex, at low levels of receptor activation [30]. Under these conditions, the higher affinity of free p85 for activated receptors allows relatively small amounts of p85 to successfully compete with heterodimeric PI3K. Furthermore, under basal conditions, the PDGFR was found to be enriched in free p85 over the p85–p110 complex [30]. Thus p85 is likely to play a role in reducing RTK signalling noise and therefore setting the threshold level for class IA PI3K activation.

### PI3K regulatory subunit dimers

Pulse-chase experiments performed on cells overexpressing p85α with or without p110α revealed a reduced half-life of free p85α [31]. Biochemical analyses suggest that surplus p85 likely exists as a dimer [32]. From here on, ‘p85 dimer’ will refer to dimers between the PI3K regulatory subunits irrespective of isoform and ‘heterodimer’ to the p85–p110 complex. Two configurations of dimeric p85 have been reported mediated by different intermolecular interactions, which will be referred to as N-terminal or C-terminal mediated dimers (Figure 1). Owing to these two possible dimer configurations, the dimeric state of p85 can be governed by several parameters, such as the cellular levels of the PI3K regulatory subunits, kinase activation or protein:protein interactions that favour one conformation.

#### N-terminal dimers of the PI3K regulatory subunits

**Figure 3.**
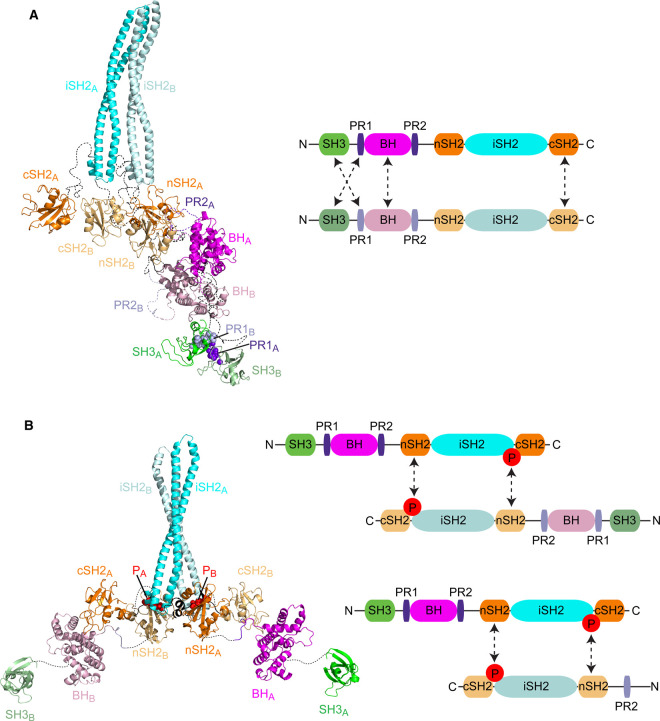
Illustrative models of PI3K regulatory subunit dimers. In each case, one monomer (named chain A) is shown in bright colours while the other (chain B) is in paler colours. The domains and regions of the proteins are labelled with a subscript to denote their chain. A key to the domain colours is shown in the schematics on the right of each model. Regions of unknown structure are indicated by dotted lines. (**A**) An N-terminal dimer model based on a combination of SAXS and cross-linking data [29]. Several models were consistent with the data but all have dimerisation interfaces between the SH3 domain of one chain and PR1 of the other chain, and between the cSH2 domains. We selected this model because a parallel orientation of the iSH2 coiled-coils was marginally favoured and there are interactions between the BH domains, which has been observed in multiple structures and shown by mutagenesis [28]. PR1 is shown as a space-filling representation. (**B**) A C-terminal dimer model based on the interaction between a phosphorylated tyrosine at the C-terminus of the iSH2 and the nSH2 domain of the other monomer. The phospho-Tyr is shown in a red space-filling representation. These dimers can be formed by two full-length regulatory subunits (p85–p85) or by the shorter isoforms (e.g. p85–p50/p55), as indicated in the schematic on the right.

p85α homodimerisation occurs both *in vitro* and *in vivo*, and in a concentration-dependent manner [33]. Intermolecular interactions between N-terminal regions of the protein can mediate dimerisation as depicted in Figures 1 and 3A; a combination of SH3 domain-proline rich motif (PR1) and BH–BH domain interactions mediate dimerisation of p85α [32–34]. Analytical ultracentrifugation studies showed that p85α undergoes rapid reversible monomer-dimer assembly and competition with a PR1 peptide blocked dimer formation, demonstrating that the SH3–PR1 interaction principally governs N-terminally mediated p85α dimerisation. The BH-BH domain contact likely also contributes to the overall dimeric interface of p85α: the same interface has been observed in multiple crystal structures although the buried surface area is rather small and the affinity of a BH-BH dimer is low [33]. Interestingly, cross-linking experiments were unable to confirm the presence of a BH dimer but rather indicated potential contacts between the C-terminal SH2 domains in the p85α homodimer [35]. p85α N-terminal dimerisation is freely reversible and the conformational states are highly heterogeneous [34]. Cross-linking data, small angle X-ray scattering data and modelling have been used to create structural models of the N-terminally mediated dimers, one of which is shown in Figure 3A [29]. Whether N-terminal dimers can form when p85 is engaged in a complex with the p110 catalytic subunit remains to elucidated but the parallel iSH2 orientation shown in Figure 3A would not support p110 interactions. Interactions between the SH3 and PR1 would not be affected by the presence of p110 and BH–BH or cSH2–cSH2 contacts could also probably still form. It is notable that dimers mediated by these N-terminal domain interactions are not applicable to the shorter isoforms, p55α, p50α and p50γ. Many cellular signalling transduction proteins utilise dimerisation as a regulatory mechanism and the PI3K regulatory subunits are no exception. In a dimeric conformation mediated principally by N-terminal domain interactions, the SH3, PR1 and possibly BH and cSH2 domains are engaged in intermolecular contacts. This conformation therefore may block binding of exogenous ligands that would otherwise engage with these domains and conversely the p85 monomer-dimer equilibrium may also be influenced by other proteins capable of interacting at these sites. For example, ligands for the SH3 domain would be competitive with the SH3–PR1 interaction that appears crucial for dimer formation. Alternatively it is also possible that a dimeric configuration may facilitate new protein:protein interactions.

Phosphatase and Tensin Homolog (PTEN), is a tumour suppressor protein and the key ‘off switch’ for the PI3K pathway. PTEN is a phosphatase that dephosphorylates the D3 position of PIP_3_, converting it back to PIP_2_ [5]. Dimeric p85α has been reported to bind PTEN, positively regulating PTEN membrane association, stability and phosphatase activity [33,36,37]. p85α associates with PTEN via the BH domain. Deletion of the iSH2 of p85 had no effect on its ability to bind PTEN, indicating that free p85 binds to PTEN [36]. The most recent report describes dimeric p85α as a PTEN stabiliser and describes the mechanism: p85α dimers compete with WWP2, an E3 ligase, for binding to PTEN protecting PTEN from WWP2-mediated degradation [33]. Engagement of PTEN into a high molecular mass PTEN-associated complex (PAC), involving p85 has also been identified and thought to promote PTEN stabilisation and activity [38]. Together these findings detail multiple cellular complexes involving p85, both independent and in complex with p110 and in different oligomeric states, all acting to regulate PTEN and subsequently signalling through the PI3K pathway.

Overall, PI3K pathway activation is influenced by the relative levels and therefore activity of p110, p85 and PTEN. The canonical role of p85 when complexed to p110 is to facilitate ligand-dependent PI3K pathway activation. However free p85 is a regulator of PIP_3_ clearance mediated by PTEN. It is likely that the monomer-dimer equilibrium of free p85 acts as gatekeeper for PI3K pathway activation and disruption to this equilibrium may result in disease. Naturally occurring somatic *PIK3R1* mutations that result in disruption of the N-terminal p85α dimer interface are frequent in endometrial cancers [15]. Indirectly, *PIK3CA* (the p110α encoding gene) amplification can also disrupt the levels of free p85α dimer as excess p110 decreases the abundance of p110-free p85α in the cell by sequestering p85α into the p85–p110 complex [39]. Therefore, the cellular levels and oligomeric state of the regulatory subunits are crucial in regulating signalling through the PI3K pathway.

#### C-terminal dimers of the PI3K regulatory subunits

Work from our own group has elucidated a new mechanism by which the PI3K regulatory subunits dimerise, regulated by interactions between C-terminal regions of the proteins (Figure 1). C-terminal dimers are instigated by phosphorylation on a conserved tyrosine residue in the iSH2 region; Tyr607 for p85α. p85α is phosphorylated downstream of the insulin receptor at Tyr607 [40] and we have found that it is directly phosphorylated by the non-RTK ACK [41]. ACK binds to all five isoforms of the regulatory subunits and phosphorylates four of them. Biochemical and biophysical analyses identified an nSH2–pTyr607 interaction, which mediated formation of p85 C-terminal dimers. The pTyr607 residue of one monomer binds to the nSH2 domain of another via a canonical SH2–pTyr binding mode (Figures 1 and 3). This particular nSH2–pTyr607 interaction is high affinity (nM) suggesting that this mechanism will be sufficient to support dimerisation independently of other interacting domains. A model structure of the nSH2–pTyr607 dimer is shown in Figure 3B, showing that symmetric intermolecular interactions can form in an antiparallel configuration. Unlike the N-terminal dimers, the pTyr607–nSH2 interaction also exists for the shorter isoforms p50α and p55α. Biochemical analysis suggests that any combination of isoforms can form heterodimers, with affinity data indicating that both p85α and p85β SH2 domains bind preferentially to p85β pTyr599 (site analogous to Tyr607 in p85α). The ramifications of such a large variety of potential C-terminal dimers remain undetermined.

p85α is processed by the ubiquitin-proteasome system (UPS) for degradation. C-terminal mediated dimers of the regulatory subunits are stabilised and protected from UPS-mediated degradation, as ACK protects wt p85α from degradation but not a non-phosphorylatable mutant Y607F p85α. In addition, ACK completely abolished polyubiquitination of p85α [41]. p42, the short isoform of ErbB3-binding protein 1 (Ebp1) is a tumour suppressor protein that targets the iSH2 region of p85α, bringing it into close proximity to its E3 ligase (the HSP70/CHIP complex) [42]. Together, these results imply that the target site for p42 is occluded in the C-terminal dimer configuration, resulting in increased p85α stability.

Cell fractionation revealed nuclear localisation of the C-terminal dimers of p85. C-terminal dimers of p50α/p85β were predominantly found in nuclear-enriched cell fractions compared with N-terminal dimers of p85α/p85β, which were cytoplasmic. Complexes of ACK and the PI3K regulatory subunits were also found in the nuclear-enriched fractions, despite the individual components showing dual localisation, and pTyr607 p85α was also predominantly nuclear [41]. The C-terminal dimers are regulated by phosphorylation and only form under conditions where ACK is active (the regulatory mechanisms and signalling pathways ACK controls have been recently reviewed [43]). Experiments using stable cell lines expressing mutant versions of p85 showed phosphorylation of p85β drives cell proliferation [41]. The functions of these C-terminal p85 dimers remain to be determined but it is likely that they have important pro-proliferative roles in the nucleus. In these dimers the nSH2 domain is engaged in the interaction with pTyr607 and therefore unable to bind pTyr residues on activated RTKs. In addition, the iSH2 region that contains the Tyr607 residue is also largely occluded in the dimer configuration and as the major site of p110 contact, it is unlikely that C-terminal dimers also engage p110. Therefore, in contrast with the canonical roles of the PI3K regulatory subunits, C-terminally mediated dimers of p85 are likely to perform previously unappreciated proliferative functions in the nucleus.

In addition to phosphorylation by ACK, other post-translational modifications play a role in regulating the functions of p85 in this region. Phosphorylation of Ser608 by the p110 catalytic subunit down-regulates PI3K lipid kinase activity [44,45]. Directly adjacent Ser609 is an IKK substrate, phosphorylation of which serves to regulate starvation-induced PI3K feedback inhibition [46]. Taken together, this suggests that residues 607–609 serve as an important regulatory region of p85 function in different cellular contexts. Additional phosphorylation sites have been identified in the nSH2 and cSH2 domains of p85α; Ser361 and Ser652, respectively [47]. These phosphorylation events within the phosphotyrosine binding pocket of the p85 SH2 domains are thought to impair PI3K activation. Sumoylation, another post-translational modification reported for p85, targets the iSH2 domain and reduced the levels of tyrosine-phosphorylated p85 [48]. The post-translational modifications described here act to modulate both p110-dependent and independent of p110 functions of p85.

Maintaining the correct balance between monomeric and dimeric p85 in a cell may be important for preventing diseases propagated by the PI3K pathway. Consequently, the regulatory mechanisms that modulate the stability and protein expression of the PI3K regulatory subunits are crucial in maintaining this balance. The stabilising effect of ACK on the PI3K regulatory subunits may underpin some of the oncogenic properties of this kinase. Unlike the standard N-terminal dimers described previously, these ACK-induced dimers have accessible N-terminal domains available for further interactions (Figure 3B) and many of the functions of free p85 require the binding capabilities of these domains. For example the proline-rich regions and BH domain have been reported to regulate the actin cytoskeleton [27,28]. The BH domain is a RhoGAP domain and has been reported to have GAP activity towards various GTPases including Cdc42 and Rac1, but most notably towards some Rab proteins, potentially making p85 a key player in receptor endocytosis [49]. The *in vitro* RabGAP activity is very weak, although GAP-deficient mutants impaired receptor trafficking through Rab4 and Rab5 in cell assays and cause cellular transformation [50,51]. The roles of the C-terminally mediated dimers in performing these critical cellular functions remain unclear but the requirement of the N-terminal domains in mediating functions of free p85 provides strong evidence for their physiological relevance.

## The nuclear functions of free p85 in stress response pathways

Studies have shown nuclear translocation of PI3K in response to various growth factor signals [52,53]. PI3K has been shown to accumulate in the nucleus of MC3T3-E1 osteoblasts in response to insulin-like growth factor (IGF-1) and platelet-derived growth factor (PDGF). This was concomitant with an increase in tyrosine phosphorylated nuclear p85 and in fact nearly all nuclear p85 was tyrosine phosphorylated under these conditions [52]. The site of this phosphorylation was not identified but could, of course, be Tyr607 on p85α, the target site for ACK, which is activated upon insulin stimulation [54]. This would fit with our recent findings of nuclear localised C-terminal dimers of p85 [41].

### The unfolded-protein response

**Figure 4.**
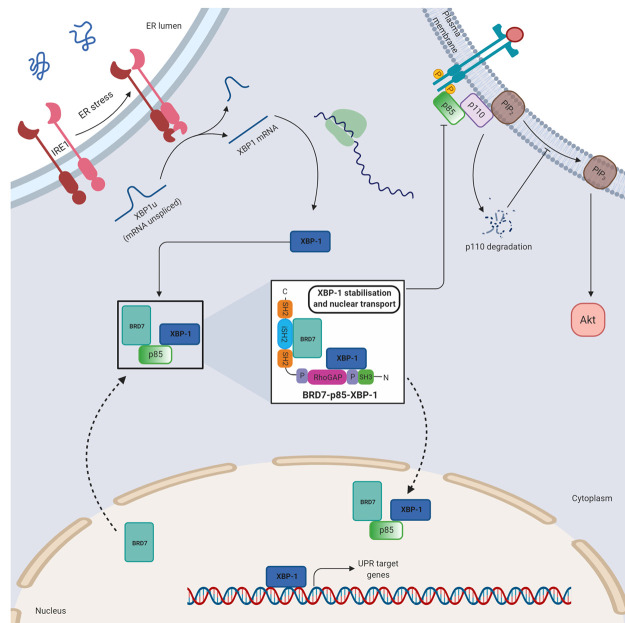
Free p85 functions in the UPR. Cartoon summarising the UPR and the known points of influence by free p85. Free p85 forms a tri-complex with BRD7 and XBP-1, facilitating the nuclear localisation of XBP-1, the key mediator of the UPR. In turn, nuclear localisation of p85 reduces cytosolic p110 and subsequently represses signalling through the PI3K pathway. BRD7, Bromodomain-containing protein 7; ER, Endoplasmic Reticulum; iSH2, inter SH2 domain; IRE1, Inositol-requiring enzyme 1; P, proline rich motif; PIP_2_, phosphatidylinositol 4,5-bisphosphate; PIP_3_, phosphoinositide 3,4,5-triphosphate; RhoGAP, Rho-family GTPase activating protein domain; SH2, Src-homology 2 domain; SH3, Src-homology 3 domain; UPR, Unfolded Response Pathway; XBP-1, X-Box binding protein 1.

The unfolded protein response (UPR) is a cascade of complex signalling networks triggered by conditions of endoplasmic reticulum (ER) stress. In response to an accumulation of unfolded or misfolded proteins in the ER the UPR is activated. X-box binding protein-1 (XBP-1), one of the main regulators of the UPR, is a transcription factor that up-regulates genes crucial in controlling the folding capacity of the ER. The PI3K regulatory subunits, p85α and p85β have been shown to interact with XBP-1, resulting in increased XBP-1 stabilisation and nuclear translocation (Figure 4). p85α knock out (KO) cells displayed alterations in the UPR, decreased induction of UPR target genes and an increased apoptosis rate [21–23]. The oligomeric state of p85 that interacts with XBP-1 is undetermined but the interaction requires the N-terminal portion of p85α. By facilitating nuclear localisation of XBP-1, the PI3K regulatory subunits mediate the cellular response to ER stress.

Bromodomain-containing protein 7 (BRD7) also plays a role in the PI3K response to ER stress and is predominantly nuclear due to its N-terminal nuclear localisation signal (NLS). Immunofluorescence analysis revealed that GFP-p85α was localised to the nucleus when co-expressed with BRD7 but was otherwise primarily located in the cytosol [55]. BRD7 binds to the iSH2 region of free p85α rather than p85 within the p85/p110 heterodimer complex and regulates its nuclear translocation. By sequestering p85 in the nucleus of cells, BRD7 indirectly reduces the level of cytosolic p110 due to its intrinsic instability and therefore attenuates PI3K signalling (Figure 4).

In agreement with its role in mediating nuclear localisation of the PI3K regulatory subunits, BRD7 indirectly controls the nuclear localisation of XBP-1 (Figure 4). An insulin-induced tricomplex BRD7–p85–XBP-1 exists, resulting in the nuclear translocation of XBP-1. Both BRD7 deficiency and p85a/β knock out reduced nuclear localisation of XBP-1. An interaction between BRD7 and XBP-1 could not be detected in cells in which the PI3K regulatory subunits had been knocked out, suggesting that p85α or p85β scaffold the BRD7-XBP-1 interaction [56]. Insulin disrupts dimerisation of p85α and p85β, promoting their association with XBP-1 and formation of the BRD7–p85–XBP-1 complex [22]. By promoting the association of XBP-1 and p85, leading to nuclear localisation of XBP-1, insulin signalling is an important mediator of the cellular stress response.

Cross-talk between PI3-Kinase signalling and the UPR cascade has critical roles in glucose homeostasis. As part of the BRD7–p85–XBP-1 tricomplex, the PI3K regulatory subunits play crucial roles in relieving ER stress, a key feature of peripheral insulin resistance and obesity. *In vivo* studies reveal that the interaction between p85 and XBP-1 is lost in obese mice. These mice exhibit ∼50% reduced expression of p85α in the liver, defects in XBP-1 nuclear translocation and heightened ER stress [56]. XBP-1 is considered to be a central player in maintenance of glucose homeostasis due to its ability to reduce ER stress, increase insulin sensitivity and direct FoxO1 degradation [22,57–59]. The ability of the PI3K regulatory subunits to regulate the nuclear localisation of XBP-1 is fundamental to its role in relieving ER stress, highlighting a role for the PI3K regulatory subunits in glucose homeostasis.

### p53-mediated cell senescence

p53 is commonly referred to as ‘the guardian of the genome’ due to its pivotal role in determining cell fate under conditions of genotoxic stress. In response to DNA damage, p53 activates transcription of numerous genes directing cell cycle arrest, senescence or apoptosis. Apoptotic cell death mediated by p53 is blocked in cells with little to no p85α expression [25]. Accumulation of reactive oxygen species (ROS) causes oxidative stress and when produced in high quantities, ROS can overload the cell's detoxification systems, disrupt metabolic pathways and cause many human diseases. Normal MEFs are sacrificed under oxidative stress generated by H_2_O_2_ but p85α KO MEFs were resistant to H_2_O_2_ treatment. Chemical inhibition revealed that the involvement of p85α in the apoptotic response was independent of PI3K catalytic activity. In response to oxidative stress, p85 therefore acts as a downstream signal transducer mediating cell death regulated by p53 [60].

**Figure 5.**
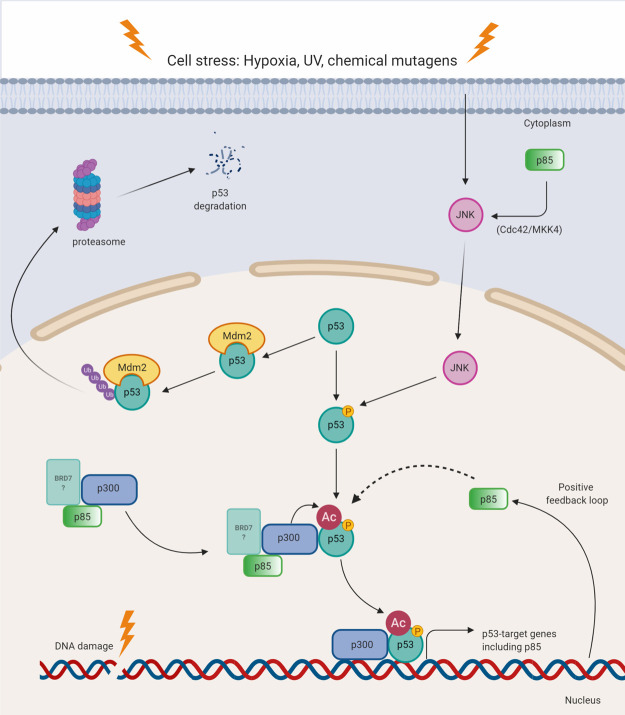
Free p85 functions in p53-mediated senescence. Cartoon summarising p53-mediated senescence and the known points of influence by free p85. Free p85 forms a complex with p300, enhancing the p53–p300 interaction and subsequent activation of p53 by acetylation. With parallel roles of BRD7 and p85 in enhancing p300-mediated p53 acetylation, the BRD7–p53 complex is speculative in this model as denoted by faint colouring and a question mark. p85α mediated activation of JNK may augment JNK driven activation of p53 by direct phosphorylation. Acetylation sites are shown as red circles and phosphorylation sites as orange circles. BRD7, Bromodomain-containing protein 7; JNK, c-Jun N-terminal Kinase; Mdm2, Mouse double minute 2 homolog; p53, transcription factor; p300, histone acetyltransferase; Ub, ubiquitin.

Analogous to its role in oxidative stress, p85α is also involved in proapoptotic responses to UV [24,61]. Post-translational modifications regulate p53 protein stability, activation and transcriptional activity. Acetylation has been described as indispensable for p53 activation, with the loss of acetylation completely abolishing p53-dependent growth arrest and apoptosis. Acetylation of p53 blocks Mdm2-mediated inhibition leading to p53 activation [62]. p85α was identified as a positive regulator of p53 acetylation in the UV-B response. p300 is a histone acetyl-transferase which acetylates the C-terminal regulatory domain of p53 under conditions of cell stress. p85α binds to p300, enhancing the p53–p300 interaction and the subsequent p300-mediated p53 acetylation. This promotes recruitment of the p53–p300 complex to promoter regions of specific p53 target genes, mediators of the pro-apoptotic UV-B response (Figure 5). In the absence of p85α, p53 was not acetylated and both the p53–p300 interaction and promoter binding capability of p53 were impaired [24]. These findings highlight free p85α as a critical upstream proapoptotic mediator in the UV-B response. Phosphorylation of p53 by JNK also blocks Mdm2 binding and targeting of p53 ubiquitination, enhancing its stability and subsequent activity [63]. p85α mediated activation of JNK [64], may attenuate this positive signal in the apoptotic response (Figure 5). In addition p85 is up-regulated by p53 at the transcription level, creating a feedback loop in which p53 up-regulates p85 expression enhancing its role in mediating p53-dependent cell senescence [25] (Figure 5). p53-mediated up-regulation of p85α further indicates critical proapoptotic roles for p85 in response to cellular damage.

Tumour necrosis factor alpha (TNF-α) is an inflammatory cytokine induced by UV and contributes to UV-induced apoptosis [65]. The nuclear factor of activated T-cells (NFAT) family of transcription factors have been shown to be key players responsible for TNF-α induction in T and B lymphocytes [66]. p85α regulates the nuclear translocation, recruitment and binding of NFAT3 to the TNF-α promoter upon UV exposure, inducing TNF-α expression [61]. TNF-α promoter-driven luciferase activities were significantly induced in wildtype MEFs in a p85 dose-dependent manner but suppressed in p85α KO MEFs. Reintroduction of p85 into null mutant cells restored the TNF-α driven luciferase signal in response to UV-B radiation. Furthermore, knock down of p85α blocked NFAT activity and subsequent TNF-α expression as well as cell apoptosis. These findings suggest p85α functions in a signalling pathway with NFAT3 and TNF-α, coordinating cellular apoptotic responses under conditions of UV stress [61]. Overall these data describe the role of free p85 as an upstream coordinator in response to cell stress [67].

Apoptosis is a crucial response to cellular damage and deregulation of apoptotic responses to genotoxic stress contributes significantly to the progress of carcinogenesis. The tumour suppressor BRD7 is deleted in many human cancers [68]. In addition to its role in regulating PI3K signalling described above, BRD7 also plays a role in p53 signalling. BRD7 has emerged as a critical regulator of p53 function required for replicative senescence [69,70]. The role of BRD7 in replicative senescence is analogous to that of p85α. BRD7 interacts with p53 and p300 and is recruited to p53-target promoters influencing p53 acetylation and therefore its activity. As BRD7 and p85α form a complex in cells [55], it is possible that they may act synergistically to mediate p53 acetylation and induce replicative senescence. The role of BRD7 in regulating p85α and XBP-1 in the UPR may mirror an undiscovered role of BRD7 in mediating p85α and p53 in the pro-apoptotic response (Figure 5). Tight regulation of p53 is imperative for maintaining normal cell growth and preventing tumourigenesis. The roles of the PI3K regulatory subunits are focal in the regulation of p53 and highlight these proteins as fundamental mediators of apoptotic pathways in response to DNA damage.

## The roles of the PI3K regulatory subunits in insulin signalling

### Class IA PI3K regulatory subunits have positive and negative roles in the insulin response

**Figure 6.**
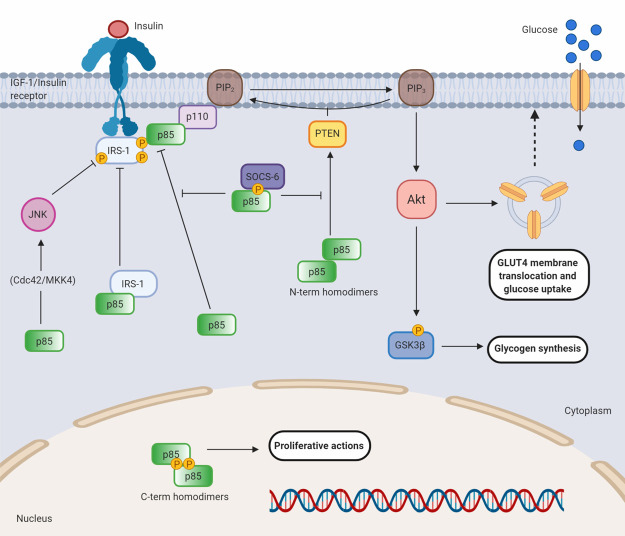
p85 roles (p110 dependent and independent) in the insulin response. Cartoon summarising the role of the PI3K regulatory subunits in the insulin response, both in heterodimer and homodimer complexes. As part of the heterodimer, p85 mediates PI3K signalling in the insulin response and subsequent activation of the Akt/mTOR pathway, initiating glucose uptake and glycogen synthesis. Free p85 suppresses insulin signalling by competing with the p85/p110 complex and IRS-1 for pTyr binding sites on activated receptors. p85 mediated activation of JNK also suppresses signalling through IRS-1/PI3K. N-terminal mediated dimers suppress insulin signalling through activation of PTEN. SOCS-6 provides a mechanism to suppress the inhibitory actions of free p85. Insulin induced C-terminal dimers have undiscovered proliferative functions in the nucleus. Phosphorylation sites are shown as orange circles. Akt, AKR mouse thyoma; Glut4, glucose transporter 4, GSK3, glycogen synthase kinase 3, IGF-1, Insulin-like growth factor 1; IRS-1, insulin receptor substrate 1; PTEN, Phosphatase and tensin homologue; PIP_2_, phosphatidylinositol 4,5-biphosphate; PIP_3_, phosphatidylinositol (3,4,5)-trisphosphate; SOCS-6, suppressors of cytokine signalling 6.

PI3K is essential for signalling through the insulin and IGF-1 RTKs, making it central to the metabolic effects of insulin including glucose uptake and glycogen synthesis (Figure 6) [71–74]. The major mechanism of activating PI3K involves docking of the insulin receptor substrate (IRS) adaptor proteins to the phosphorylated insulin receptors via their SH2 domains. Upon insulin stimulation the receptors phosphorylate IRS-1, creating p85-binding sites resulting in recruitment of PI3K and its subsequent activation. In turn the lipid products of PI3K then activate a variety of intracellular signalling pathways including Akt. Activation of the Akt/mTOR signalling pathway initiates protein synthesis and GLUT4 membrane translocation, enhancing glucose uptake.

However class IA PI3K regulatory subunits have both positive and negative roles in insulin signalling (Figure 6). While the p85 subunit is central to the metabolic actions of insulin as a component of the PI3K holoenzyme it also plays a significant role in supressing insulin signalling independently. Two mechanisms of negative regulation by free p85 act synergistically to control the amplitude and duration of the PI3K signal downstream of IRS-1. p110-free p85 competes with the p85/p110 complex for binding to IRS-1, inhibiting insulin signalling. Enhanced PI3K signalling observed in p85α heterozygous KO cells is attributed to the loss of free p85α inhibitory functions, resulting in improved PI3K mediated biological response to IGF-1[12]. Further evidence of competitive binding of free p85 is evident in cells with defective proteasomal degradation of p110-free p85β. Defects in FBXL2-mediated degradation of p85β resulted in reduced binding of p110 to IRS-1 and attenuation of the PI3K signal [75]. In addition to being a competitive inhibitor, free p85α sequesters IRS-1 in large cytosolic protein complexes blocking PI3K signalling [76]. Following withdrawal of IGF-1 the p85–IRS-1 complex exists for an extended time period and serves to attenuate persistent insulin or IGF-1 stimulation. These two mechanisms of negative regulation by free p85 are essential for regulating PI3K signalling downstream of IRS-1.

Insulin induces expression of SOCS-6, a member of the suppressors of cytokine signalling (SOCS) family. SOCS-6 offers a mechanism by which insulin can transiently overcome the negative effects of free p85 on PI3K signalling pathways (Figure 6). SOCS-6 interacts with free p85 but not the p85/p110 heterodimer upon insulin stimulation. It is also possible that SOCS-6 inhibits the up-regulation of PTEN activity by dimeric p85. This aligns with improved glucose metabolism and enhanced Akt activation, despite little increase in PI3K activity in mouse models which overexpress SOCS-6 [77]. Like the interaction with XBP-1, the interaction of free p85 with SOCS-6 is dependent upon insulin stimulation and involves the SOCS-6 SH2 domain. Several insulin-induced phosphorylation sites exist on p85 and Tyr607 is one insulin-induced phosphorylation site that conforms to the SOCS-6 SH2 binding consensus. Tyr607, located in the p110-interacting region, is likely to be inaccessible in the p85/p110 heterodimer complex, which may explain the preference of SOCS-6 for free p85. The pTyr607/nSH2 C-terminal mediated dimers of p85 may therefore compete with the binding of SOCS-6 and the equilibrium between monomeric and dimeric p85 may offer additional control of free p85 in regulating insulin signalling.

Obesity is a pathophysiological condition of heightened cellular stress including hyperinsulinemia, ER stress and inflammation. ER stress is a key feature of peripheral insulin resistance frequently found in obesity, type 2 diabetes and other metabolic syndromes. Obesity and other insulin-resistant states trigger activation of the stress kinase c-Jun N-terminal Kinase (JNK). JNK-mediated phosphorylation of IRS-1 attenuates insulin signalling through the PI3K pathway (Figure 6). As well as its role in the UPR (section 3.1), p110-independent p85α has been reported to activate JNK via the Cdc42/MKK4 pathway following insulin stimulation and ER stress. Mice with liver specific p85α deletion exhibited 75% reduced JNK activation providing evidence for the requirement of p85α for full JNK activation. These mice also showed reduced insulin-stimulated Cdc42 activity and MKK4 phosphorylation [64]. p85 has been shown to bind active Cdc42 [78] but it is unknown which isoforms constitute the p85 dimer that functions in the Cdc42/MKK4/JNK signalling node. These findings detail an additional mechanism by which p110-free p85α negatively regulates insulin signalling, further highlighting its dual role in both positive and negative regulation of the insulin response.

### Insulin sensitive mouse models reveal specificity and redundancy in the regulatory subunit isoforms

Knockdown of the PI3K catalytic subunit or the regulatory subunit have opposite effects on insulin sensitivity in mice. Both heterozygous *PIK3R1*^+/−^ and p85α KO mice displayed increase glucose uptake and insulin sensitivity. This is in contrast with *PIK3CA*^+/−^ mice that are mildly glucose intolerant and have reduced insulin sensitivity [31,79]. With a central role in insulin signalling, disruption of the PI3K regulatory subunits was expected to result in impaired PI3K signalling and an insulin phenotype similar to p110^+/−^ mice. However, the unexpected finding of increased insulin sensitivity in mice with deletion of class IA regulatory subunits has been observed in many studies [31,79–83].

*PIK3R1* encodes p85α, p55α and p50α. *PIK3R1* KO is embryonic lethal in mice, whereas selective p85α KO that retain p55α and p50α expression are viable [79,81]. Mouse models in which *PIK3R1* is knocked out have been extensively investigated for insulin-related phenotypes. Analysis revealed a complex network of interactions between isoforms of the heterodimeric PI3K complex. Targeting of regulatory subunits often alters the PI3K subunit profile in cells. Due to its intrinsic instability, p110 expression is severely reduced in *PIK3R1* null mice. Likewise, p85 is unstable in the absence of p110 [31]. The instability of p110 and p85 results in altered levels of these subunits in *PIK3R* and *PIK3C* targeted mouse models, respectively. In addition, the shorter regulatory isoforms are up-regulated in response to p85 KO [81]. Therefore, targeting a single component of the PI3K network alters the expression and protein levels of non-targeted subunits. Due to this phenomenon, mouse models often have altered expression of various components of the PI3K signalling pathway beyond those initially targeted and consequently the observed phenotype and signalling changes in these mice may not be a direct consequence of the targeted gene KO, making their interpretation complicated. This tight interplay of subunit expression suggests a level of redundancy in the PI3K signalling network.

It is not fully understood why multiple isoforms of the class IA PI3K regulatory subunits exist and/or if they have independent biological roles. The different isoforms of the PI3K regulatory subunits may offer a level of signalling specificity or represent some level of functional redundancy in the PI3K signalling network. In the insulin response, an isoform switch exists to mediate PI3K signalling despite p85α KO, so that p50α and p55α perform compensatory functions in p85α KO mice. In p85α^−/−^ mice, p50α mediates PI3K activity associated with IRS-1 [81]. Despite being N-terminally truncated, the shorter variants retain the p110 binding interface and still function in the PI3K heterodimer, offering a compensatory safety net as part of the PI3K heterodimer, mediating PI3K signalling in the face of p85α KO. In fact, p85α null mice exhibit up-regulated expression of p50α/p55α isoforms suggesting important signalling roles of the shorter isoforms in mediating insulin action [81]. Furthermore, p50α/p55α KO mice exhibit enhanced insulin sensitivity, similar to p85α KO and p85α^+/−^ mice [83]. With comparable insulin phenotypes seen in transgenic mouse models, it is likely that both the longer and shorter isoforms of p85α participate in compensatory functions to mediate the insulin response. Furthermore, a change in isoform expression occurs under conditions of cell stress. Changes in the levels of the regulatory subunits of PI3K are observed in animals with insulin resistance and diabetes. For example ob/ob mice exhibit a 50% reduction in hepatic p85α, whereas the level of p50α and p55α is increased [84]. Changes in the expression levels of the different regulatory isoforms reflects a level of redundancy in their functions as part of the PI3K heterodimer but also reflects individual roles of the different regulatory isoforms.

The mechanism of increased PI3K signalling downstream of the insulin receptor upon knock out of the class IA regulatory subunits is not fully understood. Heterozygous deletion of *PIK3R1* resulted in increased PIP_3_ production and subsequent Akt activation [12,80]. The inhibitory functions of free p85 in competing with the p85/p110 complex for IRS protein binding can partly account for the phenotypes observed. However this mechanism is not sufficient as shown by unsuccessful recovery experiments; reintroduction of increasing amounts of p85α in p85 KO adipocytes did not decrease recruitment of p85/p110 heterodimers or insulin-stimulated PI3K activity [72,85].

Increased Akt activation is frequently observed in p85 KO cells despite inconsistent changes in PI3K activity. Akt activation is an indirect measurement of the level of cellular PIP_3_. As the level of PIP_3_ is controlled by two antagonistic pathways: PI3K lipid kinase and PTEN/SHP-2 phosphatase activity, direct stimulation of PTEN activity by free p85 satisfies the paradox identified in the regulation of insulin signalling by p85 and p110 [26,36]. Improved insulin sensitivity in the p85α^−/−^ and *PIK3R1*^+/−^ mice may be attributed to reduced positive regulation of PTEN by dimeric p85. Reduced PTEN activity would sustain PIP_3_ levels even in the face of unchanged PI3K activity, resulting in enhanced Akt activation. In accordance with this, mice with liver-specific p85α KO have reduced PTEN phosphatase activity, resulting in reduced PIP_3_ clearance and enhanced Akt activation [26]. In addition, PTEN^+/−^ mice show the same phenotype of increased insulin sensitivity as *PIK3R1*^+/−^ and p85α^−/−^ mice, indicating parallel roles of PTEN and p85α in coordinating the insulin response [86,87]. Although homodimerised p85α is a known positive regulator of PTEN stability and activity, the exact conformation of the p85α dimer that regulates PTEN has not been established, however this activity does require the BH domain of one monomer [33]. Overall therefore, p85 is a dual regulatory protein for both PI3K and PTEN, performing critical roles in order to maintain the balance of PI3K/PTEN signalling in the insulin response [88].

Typically, p110-independent functions of the regulatory subunits rely on the N-terminal domains of p85. The shorter isoforms are N-terminally truncated and as a result cannot fully compensate for all functions of the p85 isoforms in the insulin response. Activation of JNK could not be reconstituted with re-expression of p55α or p50α in mice with liver specific p85 KO [64]. Similarly, the N-terminal BH and SH3 domains of p85 are required for the interaction with PTEN, suggesting that the shorter isoforms will be unable to fully compensate in the p85/PTEN signalling node [33]. p110-independent functions conducted by the longer isoforms are good evidence of specificity amongst the PI3K regulatory subunits in the insulin response. The requirement for the N-terminal domains of the PI3K regulatory subunits in mediating the p110-independent functions of p85 in the insulin response also provides strong evidence for the physiological relevance of C-terminal dimers, in which the N-terminal protein domains are freely accessible.

p110-independent functions of p85 are likely to be performed by dimeric p85. Targeted *PIK3R* gene KO likely disrupts the dynamic equilibrium between monomeric and dimeric p85 (and of homo-dimeric and hetero-dimeric p85 species), which may impact the critical functions of free p85 in the insulin response. With the compensatory roles of p85 isoforms in PI3K signalling and specificity reflected in the p110-independent functions of p85 mediated by its N-terminal domains, care has to be taken when interpreting phenotypes of transgenic mouse models. The cellular levels of the different regulatory subunit isoforms are crucial in regulating signalling through the PI3K network and disruption to this balance will impede glucose homeostasis.

## PI3K-independent roles of the class IA PI3K regulatory subunits in receptor trafficking and cell migration

### GAP activity of the PI3K regulatory subunits towards the Rab GTPases controls receptor down-regulation

**Figure 7.**
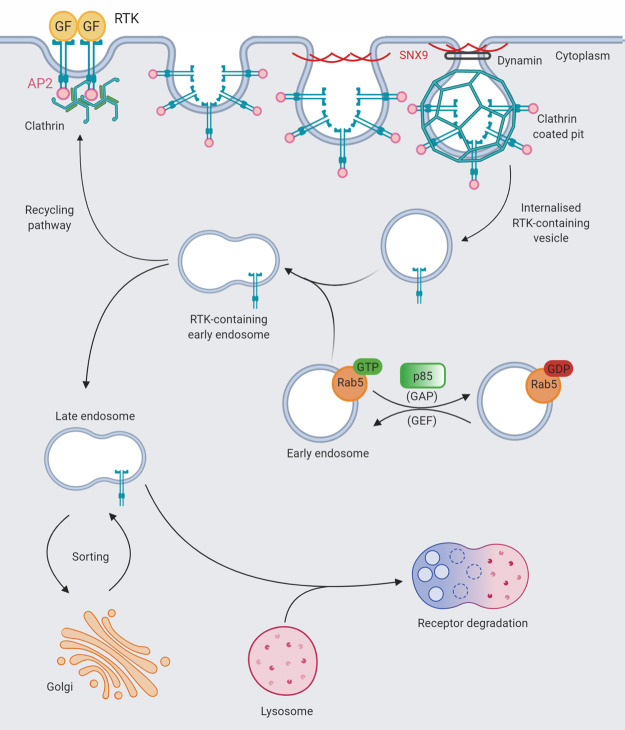
Schematic of Rab5 mediated endosomal trafficking in receptor down-regulation and influences by free p85. Cartoon summarising endosomal trafficking and the known points of influence of free p85. The ligand-bound receptor is internalised within an intracellular clathrin lattice. The clathrin lattice invaginates forming a clathrin-coated pit that pinches off to form a clathrin-coated vesicle. This intermediate vesicle fuses with the early endosome, delivering the receptor. This vesicle fusion event is mediated by Rab5 that localises to early endosomes. p85 GAP activity towards Rab5·GTP switches off this initial fusion event and is implicated in receptor down-regulation. The RTK-containing early endosome can also be trafficked through the endocytic pathway to the lysosome for degradation. AP2, adaptor protein2; GF, growth factor; SNX9, sorting nexin 9; GAP, GTPase activating protein; GEF, guanine nucleotide exchange factor.

Eukaryotic cells use endocytic pathways to modulate plasma membrane content and therefore regulate the steady-state level of receptors, ligands and associated factors at the cell surface. Endocytosis of RTKs results in internalisation, transportation, sorting and degradation (Figure 7). Following stimulation, the cell surface invaginates and the RTK is recruited to clathrin-coated pits, which are internalised into the cell. Vesicles containing internalised receptors and associated proteins are trafficked through the endocytic pathway to lysosomes for degradation. The importance of the PI3K regulatory subunits in receptor down-regulation is evident in cells expressing mutant PDGFR that cannot be phosphorylated at the p85-binding residues (Y740F and Y751F), which despite being internalised failed to be trafficked to the lysosome for degradation [89,90]. The PI3K heterodimer remains associated with activated RTK-containing vesicles and performs critical regulatory roles to mediate vesicle trafficking.

Trafficking of RTK-containing vesicles is regulated by the Rab family of monomeric small GTPases, which control vesicle fusion events [91]. Rab5 regulates fusion events during endocytosis of activated RTKs, important for transportation of the receptor complex from the plasma membrane to early endosomes. Rab4 regulates vesicle trafficking from early endosomes back to the plasma membrane in the recycling of receptors [92]. The GTP-bound Rab proteins localise to early endosomes, binding to the early endosome autoantigen 1 (EEA1) to mediate membrane fusion events. Rab·GDP cannot bind EEA1 making the GTPase activity of Rab4 and 5 the rate-limiting component of early endocytic fusion events [91,93]. The roles of Rab5 and Rab4 in regulating trafficking of internalised RTK complexes via the endocytic pathway, in turn control the subcellular localisation, magnitude and duration of receptor signalling.

p85α and p85β contain a Bcr Homology domain (BH), which is homologous to the RhoGAP (Rho family GTPase Activating Protein) domain of Bcr and has been implicated in regulating a complex network of GTPases. p85α can bind Cdc42, Rac1 and Rab GTPase family members [49,78,94] and has been reported to exert GAP activity towards Rab5, Rab4, Cdc42 and Rac1, albeit at very low levels [49]. The GAP activity of p85α targeted to Rab5 has been implicated in the regulation of receptor down-regulation (Figure 7). p85α was found to bind Rab5 in a low affinity interaction, independent of its nucleotide-bound state and stimulated the hydrolysis of GTP 100-1000 fold, in a concentration dependent manner *in vitro*. The low level of activity and nucleotide independency of this interaction is uncharacteristic for a GAP. Typically, GAPs provide two functions that enhance the intrinsic GTP hydrolysis of GTPases: they stabilise the switch regions of the GTPase and they provide a catalytic arginine residue to stabilise the transition state during GTP hydrolysis. Arg274 was implicated as the arginine finger in p85α and cells expressing R274A p85α displayed impaired receptor degradation, resulting in sustained MAPK and Akt signalling. R274A p85α could still bind p110 and did not affect PI3K activity in response to PDGF stimulation, suggesting the effects of this mutant are independent of PI3K activity [51]. Further work has explored the kinetics of impaired receptor down-regulation in this mutant [50]. In NIH3T3 cells, expression of R274A p85α resulted in cellular transformation, which was reversed by the expression of a dominant negative Rab5 [51], indicating the lack of GAP activity towards Rab5 in the R274A p85α mutant as the cause of cellular transformation.

The involvement of specific dimeric forms of free p85 in vesicular trafficking and receptor down-regulation is presently unclear. The BH/RhoGAP domain resides in the N-terminal half of p85 but is accessible in both N-terminally and C-terminally mediated dimers and it is tempting to speculate that its activity is modulated by dimer formation. For example, in the dimers the GAP activity of p85 could be increased due to a higher, localised concentration of the p85 subunit. Alternatively, the conformation of the BH domain could change in either a C-terminal or N-terminal dimer configuration, which may alter its GAP activity. Modified GAP activity of the BH domain in a p85α homodimer would affect Rab-mediated endocytic trafficking. There is currently no data on the modulation of the BH domain GAP activity, by either direct or indirect mechanisms, so the effects of dimerisation are still unclear.

The findings discussed here detail a PI3K independent mechanism of p85α in mediating receptor down-regulation. Through the temporal control of the GTPase cycles of Rab4 and Rab5, p85α plays a fundamental role in the regulation of receptor sorting functions and down-regulation, disruption of which contributes to tumourigenesis.

### The role of p85 in mediating Cdc42-induced cytoskeleton changes

The cytoskeleton is a network of fibres providing shape and support to the cell, fulfilling an organisational role and facilitating cargo transport. Cell migration utilises cytoskeletal rearrangements as its driving force. Cells initiate migration by polarising and extending protrusions of the cell membrane, known as filopodia, towards external cues [95]. The small G proteins of the Rho family play essential roles in regulating the actin cytoskeleton [96]. Cdc42, a member of the Rho family along with its effector Wiskott–Aldrich Syndrome Protein (WASP) control the co-ordinated assembly and disassembly of actin filaments in filopodia formation [97]. p85α was found to enhance Cdc42 activation and trigger Cdc42-dependent cytoskeletal changes in response to PDGFR stimulation. These changes included filopodia formation, decrease in actin stress fibres and a reduction in focal adhesion complexes, all of which occurred independently of PI3K enzymatic activity [27]. Stress fibres are highly regulated actomyosin structures that play important roles in cellular contractility and provide the force for cell adhesion and migration. A mutant PDGFR that could not bind p85α was unable to stimulate the disassembly of stress fibres. This was again independent of PI3K activity as wortmannin treatment did not prevent stress fibre disassembly induced by wildtype PDGFR. Along with cell contractility, Cdc42-triggered disassembly of focal adhesion complexes also underpins cell migration. Focal adhesion complexes provide the main site of cell adhesion between the cytoskeleton and extracellular matrix, with their co-ordinated assembly and disassembly necessary for cell migration [98,99]. The regulation of focal adhesion disassembly by p85α plays a role in cell migration. In agreement with this, p85α^−/−^ bone marrow-derived macrophages demonstrated a severe reduction in migration compared with wildtype cells and reduced cell adhesion [100]. p85β has also been found to trigger Cdc42 regulated cytoskeletal changes. By recruiting active Cdc42 (and Rac1) to cell adhesion contacts, p85β regulates invadopodium formation [101] and the metastatic potential of tumours with increased p85β expression may in fact be explained by p85β co-ordinated invadopodium extensions. These findings demonstrate a role for p85 in mediating Cdc42-induced cytoskeletal changes.

The function of p85 in cytoskeletal remodelling is also an important component of cytokinesis and represents another PI3K independent role of the PI3K regulatory subunits [102]. Cytokinesis requires cooperation of cytoskeletal components such as actin, myosin and septin, and the Rho family GTPases regulate the actin cytoskeleton during cytokinesis. p85-regulated Cdc42 activation is an essential event in cytokinesis as shown by the accumulation of binucleated and telophase cells deficient in p85 [103]. Cytokinesis could be restored in these cells by the expression of a p85α mutant unable to bind the catalytic subunit, highlighting the p110-independence of this mechanism. p85 acts as an adaptor protein, binding to Cdc42 and septin 2 (a major driver of cytokinesis) and regulating their local concentrations at the cleavage furrow [103].

The N-terminal SH3, proline rich, BH domain region of p85 is responsible for p85-regulated cytoskeletal changes as demonstrated by the inability of the shorter variants, p50α and p55α, to compensate in stress fibre dissociation [27]. The N-terminal domains have been linked to multiple components of the cytoskeleton and therefore facilitate the adaptor function of the PI3K regulatory subunits in mediating cytoskeleton changes. The SH3 domain of p85α has been reported to bind sorting nexin-9, p130Cas, Focal Adhesion Kinase (FAK) and dynamin, all proteins associated with cytoskeleton regulation [104–108]. In addition, α-actin co-precipitated with p85 from NIH3T3 cell lysates [109]. The BH domain mediates binding of p85α to both Cdc42 and Rac1 and albeit at low levels was found to have GAP activity towards these important regulators of actin structures [49,78,110]. GAP activity was observed at micromolar concentrations of p85, however it is unknown whether these concentrations are physiologically relevant and would be incompatible with a stimulatory role towards Cdc42. Instead p85 likely acts as a scaffold protein controlling Cdc42 localisation, activation and subsequent Cdc42-driven cytoskeleton changes. Again, the dimeric configuration of p85 may modulate its adaptor capabilities. The monomer-dimer equilibrium may itself of course be influenced by favourable interactions with N-terminal domains, such as those to cytoskeleton components, providing a mechanism whereby the cytoskeleton and its regulators could influence other cellular processes.

## Conclusion

As part of the PI3K heterodimer the regulatory subunits mediate signalling through the PI3K pathway. Yet with surplus p85 existing in mammalian cells, the role of free p85 has been the focus of increased investigations. p85 and p110 are obligate partners in the cell, intrinsically instable without one another and bound with high affinity. Other binding partners that can compensate for p110 however, can stabilise free p85. Various cellular complexes containing the PI3K regulatory subunits have been reported which include but are not limited to; the canonical p85/p110 heterodimer, dimers of free p85 (N-terminally mediated and C-terminally mediated), p85/PTEN and p85-associated PAC. The availability and affinity for free p85 governs the cellular concentration of each p85-associated complex, with hierarchical binding being possible. Once all p110 is saturated with p85, excess p85 is available to bind to its other partners. N-terminal dimers are mediated by intermolecular interactions and therefore are a result of the local concentration of p110-independent p85. C-terminal dimers are triggered by phosphorylation and form following external stimuli eliciting a new signalling cascade. A dimeric conformation of the PI3K regulatory subunits may facilitate new protein interactions, as is the case for the positive regulation of PTEN by p85α homodimers. Alternatively, a dimeric configuration may block other binding interactions. Binding to pTyr residues on activated RTKs is likely modulated in the C-terminal dimers as the nSH2 domain is occupied in this configuration. Interactions that favour a particular dimer conformation will influence the monomer-dimer equilibrium of p85. Therefore, the balance of free p85 signalling complexes is crucial in regulating signalling through the PI3K pathway.

p85 performs critical adaptor functions influencing the localisation of key signalling proteins, indirectly mediating their activity. In response to cellular damage p85 plays a proapoptotic role, mediating TNF-α expression and triggering p53-mediated cell senescence. Likewise, under conditions of cell stress free p85 mediates nuclear translocation of XBP-1 making it a critical regulator of the UPR. As an adaptor, p85 functions as the intermediary between ER stress and glucose homeostasis. In the insulin response the PI3K regulatory subunits have both positive, as part of the PI3K heterodimer, and negative roles, independently of PI3K catalytic activity. Free p85 has multiple mechanisms to control the cellular level of PIP_3_/Akt activation and largely functions to set a threshold level for PI3K activation. Free p85 suppresses signalling through PI3K by occupying pTyr residues on activated RTKs and competing for IRS-1 binding. Dimeric p85 positively regulates PTEN stability and activity, reducing the levels of cellular PIP_3_. The N-terminal domains of p85 are largely responsible for many of the adaptor functions of free p85, both in the insulin response with the BH and SH3 domain mediating the PTEN interaction but also in other contexts. The BH domain binds various GTPases, including Cdc42, Rac1, Rab4 and Rab5 and the GAP activity of p85α towards Rab4 and Rab5 has been implicated in endocytic trafficking, vesicle sorting and receptor down-regulation. Localised activation of Cdc42, mediated by the adaptor capabilities of free p85 function in cytoskeleton changes driving cell migration and cytokinesis but also in JNK stress kinase activation. Activation of JNK via the Cdc42/MKK4 pathway by p85α inhibits insulin signalling and represents another example of cross-talk between PI3K signalling and cellular stress responses. A dimeric configuration of p85 may alter the accessibility and/or ability of N-terminal protein domains to perform their cellular regulatory functions. With different models of the PI3K regulatory subunit dimers and many factors influencing their local concentrations, the complexity of the PI3K signalling network cannot be underestimated.

## Perspectives

1. Importance of the field: The class IA PI3K regulatory subunits can exist independently of p110 and undertake critical functions independent of PI3K catalytic activity. A further understanding of the roles of p110-independent p85 are paramount in understanding PI3K both in homeostasis and disease.
2. Current thinking: Free p85 is dimeric, with two possible conformations reported that are mediated by N-terminal and C-terminal protein domain interactions, respectively. The functions of free p85 predominate in cellular stress pathways, yet the precise roles of dimeric p85 have not been fully elucidated.
3. Future directions: We are still a long way from understanding the full extent of the roles of free p85 in signalling pathways and how, when deregulated, this can lead to disease pathologies. In order to be able to develop safe and effective therapies targeting the PI3K pathway, a full understanding of the complex signalling networks orchestrated by the p85 regulatory subunits will be imperative.

## Open Access

Open access for this article was enabled by the participation of University of Cambridge in an all-inclusive *Read & Publish* pilot with Portland Press and the Biochemical Society under a transformative agreement with JISC.

## Abbreviations

BH: bcr-homology domain
ER: endoplasmic reticulum
GAP: GTPase activating protein
GDP: guanine nucleotide diphosphate
GTP: guanine nucleotide triphosphate
KO: knock out
PAC: PTEN-associated complex
PIP_2_: phosphatidylinositol 4,5-bisphosphate
PIP_3_: phosphoinositide 3,4,5-triphosphate
PR: proline rich motif
PROS: *PIK3CA*-related overgrowth spectrum
RhoGAP: Rho-family GTPase activating protein domain
ROS: reactive oxygen species
RTK: receptor tyrosine kinase
SAM: sterile alpha motif
SH2: Src-homology 2 domain
SH3: Src-homology 3 domain
UPR: unfolded protein response
UPS: ubiquitin-proteasome system
